# Multilevel drivers and constraints of shifts from injecting to smoking opioids and stimulants: A qualitative study from the San Diego-Tijuana border region

**DOI:** 10.1016/j.drugalcdep.2026.113279

**Published:** 2026-07-28

**Authors:** William H. Eger, Maia H. Hauschild, Erika L. Crable, Logan A. Hehner, Katie Bailey, M. Gudelia Rangel, Alicia Harvey-Vera, Carlos Vera, Annick Bórquez, Heather A. Pines, Eileen V. Pitpitan, Thomas L. Patterson, Steffanie A. Strathdee, Angela R. Bazzi

**Affiliations:** a School of Social Work, San Diego State University, San Diego, CA, USA; b School of Medicine, University of California San Diego, La Jolla, CA, USA; c Miller School of Medicine, University of Miami, Miami, FL, USA; d Fielding School of Public Health, University of California Los Angeles, Los Angeles, CA, USA; e Center for Health Policy Research, University of California Los Angeles, Los Angeles, CA, USA; f Department of Psychology, Hartwick College, Oneonta, NY, USA; g Herbert Wertheim School of Public Health, University of California San Diego, La Jolla, CA, USA; h Department of Population Studies, Colegio de la Frontera Norte, Tijuana, Mexico; i School of Public Health, San Diego State University, San Diego, CA, USA; j Boston University School of Public Health, Boston, MA, USA

**Keywords:** Fentanyl, Substance Abuse, Intravenous, Smoking, Methamphetamine, Analgesics, Opioid, Drug Overdose

## Abstract

**Background:**

Amidst declines in injecting opioids and stimulants across North America, we sought to explore multilevel drivers and constraints of shifts from injecting to smoking these substances among people with a history of injection drug use.

**Methods:**

From October-December 2024, we conducted semi-structured qualitative interviews with individuals with histories of injection drug use in San Diego, California, and Tijuana, Baja California. Eligibility included past-month heroin, fentanyl, or methamphetamine use by injection and/or smoking. Informed by the social ecological model, we used thematic analysis to examine the multilevel determinants of shifts in routes of drug use.

**Results:**

Among 36 participants (50% in San Diego), median age was 40 years, 61% were male and 58% were unsheltered. In the past month, 81% both injected and smoked heroin, fentanyl, and/or methamphetamine, 17% smoked only and 3% injected only. Rather than complete substitution, most participants described smoking as their primary route alongside intermittent or situational injection, driven by: (1) desires to avoid fentanyl-related injection harms; (2) greater ease, discretion, and lower stigma of smoking; and (3) drug-supply conditions and fentanyl–methamphetamine co-use. Sustained movement toward smoking was constrained by (4) embodied injection practices and beliefs about desired effects or withdrawal relief and (5) inconsistent access to smoking equipment.

**Conclusion:**

Individual shifts toward smoking were often intentional (e.g., to reduce health harms) but depended on drug market conditions and material environments. Expanding access to smoking equipment while maintaining syringe coverage for ongoing injection may reduce harms in the context of fentanyl–methamphetamine co-use.

## 1. Introduction

The expansion of unregulated fentanyl and methamphetamine into the North American drug supply has ushered in an era of harms, including nearly one million overdose deaths over the past decade (Friedman and Shover, 2023; Ciccarone, 2021). Although overdose deaths through 2022 were increasingly driven by polysubstance use involving fentanyl and stimulants (predominantly methamphetamine), recent national data suggest meaningful declines in overall overdose mortality (Anderer, 2025). However, deaths involving stimulants alone continue to rise, and fentanyl still contributes to a majority of overdose fatalities (Friedman et al., 2026). These patterns reflect a multifaceted risk environment for people who use drugs, particularly individuals consuming unregulated opioids and stimulants.

Alongside shifts in the unregulated drug supply, drug consumption practices have also changed markedly across North America. Beginning with reports from San Francisco, California, in 2021, researchers documented large declines in injecting heroin alongside increases in fentanyl smoking driven by individual desires to reduce injection-related harm (Kral et al., 2021). Subsequent quantitative studies revealed that this shift in behavior was not isolated to San Francisco but occurred across North America (Karandinos et al., 2024; Parent et al., 2021; Lipira et al., 2026). Qualitative research from San Francisco and Vancouver, British Columbia, further documented that shifts from injecting to smoking opioids were often motivated by health complications from injecting, efforts to regain control over consumption practices in the fentanyl era, and relative ease and discretion of smoking compared to injecting (Bonn et al., 2025; Ciccarone et al., 2024). While much literature to date has focused on declines in opioid injection, evidence from our research in the San Diego-Tijuana border region (SDTBR) suggests these shifts may also be accompanied by declines in methamphetamine injection (Eger et al., 2024a). Yet, despite its contribution to overdose risk, little is known about how methamphetamine use influences these transitions.

While prior qualitative studies have primarily examined drivers of recent shifts from injecting to smoking opioids, less is known about why some people who inject drugs (PWID) have not shifted toward smoking amidst these population-level changes in routes of administration and how co-use between fentanyl and methamphetamine may impact these shifts. To address these gaps, we conducted a qualitative study among people with a history of injection drug use enrolled in a longitudinal cohort study in the SDTBR, where recent evidence demonstrated notable declines in injecting alongside increases in smoking heroin, fentanyl, and methamphetamine (Eger et al., 2024a). Our aim was to identify multilevel drivers and constraints shaping routes of administration among PWID, including shifts toward smoking. Understanding both drivers and constraints is essential for designing interventions that not only motivate but also enable transitions away from injecting, which could meaningfully reduce injection-related harm (Bridge, 2010). Our setting at the SDTBR may further illuminate reasons for regional differences in how routes of administration have evolved across North America (Karandinos et al., 2024), given stark cross-border variation in harm reduction infrastructure (Stone and Shirley-Beavan, 2018), including the density of syringe services programs (SSPs) and paraphernalia laws (Davis et al., 2026). Using the social ecological model (SEM) (Bronfenbrenner, 1979), we examined individual, interpersonal, community, and structural factors impacting current route decisions and reported movement toward smoking heroin, fentanyl, and methamphetamine in this context.

## 2. Methods

### 2.1. Study design and sample

From October–December 2024, we conducted semi-structured interviews with a purposive subsample of participants enrolled in the ongoing *La Frontera* cohort study in San Diego County, California, and Tijuana, Baja California, Mexico. As described elsewhere (Strathdee et al., 2021), *La Frontera* examines overdose, HIV, and hepatitis C virus among PWID in the SDTBR; parent-study eligibility included age ≥ 18 years, English or Spanish fluency, residence in San Diego County or Tijuana, and past-month injection drug use. For this qualitative sub-study, we purposefully recruited participants at their follow-up visits who reported past-month heroin, fentanyl, or methamphetamine injection and/or smoking at screening (Palinkas et al., 2015); we aimed to recruit participants who injected and smoked, only smoked, and only injected. This approach was designed to capture diverse patterns in routes of administration to characterize motivations and potential limitations regarding the shift from injecting to smoking (Eger et al., 2024a). All study activities and materials were available in English and Spanish based on participants’ preferences, via trained bilingual study staff. All participants provided written informed consent and received $25 for qualitative interviews. UC San Diego and Universidad Xochicalco approved all study activities.

### 2.2. Procedures

We developed a semi-structured interview guide by reviewing existing literature and drawing from team expertise (Kral et al., 2021; Bonn et al., 2025; Ciccarone et al., 2024). Questions spanned each level of the SEM (Bronfenbrenner, 1977), including individual (e.g., “*Can you walk me through the process of how you typically consume [heroin, fentanyl, and/or methamphetamine], like through injecting, smoking, or both?*”; “*Can you explain why you tend to consume [drug] in the way you described?*”), interpersonal (e.g., *“How does your decision to inject or smoke depend on the people you are with?”*), community (e.g., “*What challenges do you have with each method of consumption when you are using in public settings?*”), and structural levels (e.g., “*How does your decision to inject or smoke depend on the drug use equipment you have available?*”). Questions were developed in English and translated to Spanish by bilingual research staff. Researchers trained in graduate-level qualitative methods conducted each interview in the preferred language of the participant (English or Spanish), and afterwards the first author iteratively took notes to identify potential emergent themes, refine the interview guide, and update the sampling strategy to ensure inclusion of diverse perspectives. We continued recruitment until we identified no substantively new conceptual insights relevant to the study aims during regular team discussions. Interviews were conducted in a private space (research team-leased office spaces or mobile vans) and lasted approximately 60–90 min. Interviews were audio-recorded, professionally transcribed (and, when needed, translated from Spanish to English by bilingual staff), reviewed for quality, and de-identified prior to analysis.

### 2.3. Data analysis

We used reflexive thematic analysis following a six-phase approach (Braun and Clarke, 2006, 2022). We first familiarized ourselves with the data by reviewing audio recordings and interview transcripts, documenting our initial observations and reflections through narrative memos (Birks et al., 2008). We then used these memos to develop a preliminary codebook, which we refined through an iterative process of double coding by two qualitative researchers (WHE and MHH) to ensure shared understanding of the coding process. We discussed coding discrepancies collaboratively with a third author (LAH) to clarify conceptual boundaries and to strengthen analytic coherence. After double-coding approximately 20% of transcripts, the first author then coded the remaining transcripts, with ongoing discussions among analysts. Following coding, WHE and MHH independently reviewed coded passages to develop preliminary themes. Theme development was primarily inductive; however, because SEM informed the interview guide and study aims, it served as a sensitizing framework during interpretation by encouraging consideration of how multilevel determinants shaped participants’ routes of administration. We remained open to patterns that did not align neatly with predetermined SEM levels. We iteratively refined themes through reflective discussions with LAH before naming and defining the final set of themes with the senior author. We then used SEM to examine relationships within and across the final themes, including how determinants operating at different SEM levels reinforced, interacted with, or constrained one another, and organized them into an analytic narrative (Bronfenbrenner, 1977). The analytic team has extensive experience conducting community-based research with PWID, informed by harm reduction principles and volunteer work with SSPs. We used memoing and regular analytic discussions to reflect on how our prior experiences could shape interpretation and remained attentive to findings that challenged our expectations. NVivo 14 was used to manage data [22].

## 3. Results

### 3.1. Sample characteristics and substance use behaviors

We interviewed 36 participants, including 18 from San Diego and 18 from Tijuana (Table 1). Median age was 40 years (interquartile range: 34, 51), 61% were male, 58% were Hispanic, Latino, or Mexican, 72% earned less than $500 USD per month, 67% had a history of incarceration, and 58% were unsheltered in the past six months. In the past month, 29 (81%) injected and smoked heroin, fentanyl and/or methamphetamine, six (17%) smoked-only, and one injected-only (3%); 58% injected heroin, 33% injected fentanyl, and 61% injected methamphetamine, while 56% smoked heroin, 50% smoked fentanyl, and 92% smoked methamphetamine. Participants last injected a median of 6 days before the interview (IQR: 0, 30), whereas smoking was typically reported the day of.

Although most participants described mixed route use, site patterns differed. In San Diego, participants more often described fentanyl and methamphetamine smoking as routine—“*all day…like every hour*” (age 34)—with injection framed as occasional or situational. Fentanyl injection was described or perceived as rare, as noted by one participant: “*I don’t really know people that inject fentanyl.”* (San Diego, age 41) In Tijuana, daily opioid injection remained more common, while “*crystal [methamphetamine] is smoked or inhaled*.” (age 30) Across sites, intermittent methamphetamine injection persisted even among participants who otherwise preferred smoking.

### 3.2. Thematic findings

Participants often described smoking as their primary route: (Friedman and Shover, 2023) to avoid individual-level harms of injection in the fentanyl era; (Ciccarone, 2021) because smoking was perceived as easier, more discreet, and less stigmatized within interpersonal networks; and (Anderer, 2025) due to structural drug supply dynamics and fentanyl–methamphetamine co-use, which drove substance-specific and situational route decisions (e.g., intermittent methamphetamine injection). However, sustained shifts toward smoking were constrained by: (Friedman and Shover, 2023) individual injection practices and perceptions about the necessity of injection to achieve desired effects or withdrawal relief; and (Ciccarone, 2021) structural and community-level gaps in access to smoking equipment, which sometimes precipitated injection.

#### 3.2.1. Individual desires to avoid overdose and injection-related harms in the fentanyl era motivated shifts toward smoking

Participants often described smoking fentanyl as sufficient to achieve desired effects without the immediate risks they associated with injection, such as overdose, skin and soft tissue infections, and vein damage. Many participants felt that *“it just doesn’t seem necessary to inject, and it’s such a huge health risk to shoot [inject], that’s why I smoke more.”* (San Diego, age 29) For some, fears surrounding fentanyl injection were so pronounced they avoided injecting fentanyl altogether. As one participant explained:

“Interviewer: Why not inject fentanyl?

Participant: Because it’s easier to O.D. [overdose] and die.

Interviewer: So, to not overdose, you started smoking it?

*Participant: Yeah*.”

(San Diego, age 31)

Participants contrasted the immediate harms of injecting with the potential longer-term respiratory risks from smoking:

“There are things you can do… you can smoke more [and] put off the health risks for later…But at the moment, you’re about to lose your arms and limbs or die from an infection because you won’t stop shooting… the person has to chill on the shooting…Knowing that, I started smoking more and shooting less.”

(San Diego, age 29)

Participants who previously injected heroin and methamphetamine frequently described reduced venous access, which increased the practicality of smoking: *“All that’s left are scars on my hands, brown marks like cuts. I struggle, poking and poking… I barely have any veins left, so I’m mostly left with smoking.”* (Tijuana, age 32) While reduced venous access was not always described as a direct consequence of fentanyl injection, participants explained that fentanyl’s potency made smoking a practical alternative to injecting because it produced the desired effects without requiring intravenous administration.

#### 3.2.2. Interpersonal and structural stigma positioned smoking as easier, more acceptable, and less harmful than injecting

Participants described smoking as increasingly normalized within their social networks, whereas injecting was viewed as “*the worst*”, particularly by younger people. (Tijuana, age 34) Smoking, on the other hand, was framed as *“a lot more accepted.”* (San Diego, age 34)

Stigma toward injection drug use was further intensified by the perceived dangers of injecting fentanyl, with concerns about overdose extending beyond participants’ own safety to shape how others viewed and responded to fentanyl injection. For example, one participant explained that suppliers routinely discouraged fentanyl injection because *“they don’t wanna be responsible for you OD’ing [overdosing] and dying,”* instead warning customers, *“Don’t shoot this. You’ll die.”* (San Diego, age 59) These concerns appeared to reinforce social norms that discouraged injecting fentanyl in shared settings and contributed to perceptions that injection had become an increasingly private behavior: *“injecting is more private. You can’t just do that anywhere.”* (Tijuana, age 39) In contrast, smoking was described as more social, quicker, and easier to conceal in public spaces. This distinction was especially salient for participants experiencing unsheltered homelessness, for whom avoiding police contact was a daily concern. One participant noted how *“a cop driving by seeing someone shooting up is a lot more likely to pull over, as opposed to seeing someone taking a hit [smoking].”* (San Diego, age 41)

Additionally, participants said that visible signs of injecting could expose them to stigma and criminalization, positioning smoking as more socially acceptable and safer.

#### 3.2.3. Structural drug market dynamics and polysubstance use drove situational shifts in routes of administration

Drug supply conditions shaped route decisions in individualized rather than uniform ways. Several participants whose *“drugs of choice are heroin and cocaine”* (San Diego, age 41) said when those drugs were unavailable or unaffordable, they used the substances they could obtain—most often fentanyl and methamphetamine. As noted above, for most, this meant smoking fentanyl despite preferring to inject heroin because “*the [fentanyl] is so strong, there’s just no need to shoot [inject] it*” (San Diego, age 29). Thus, some participants who preferred injecting heroin smoked fentanyl when heroin was unavailable.

For others, the superior intensity and efficiency of injecting (compared to smoking) was a key reason they continued to inject intermittently. In this way, participants adapted their route of administration to achieve their desired potency and effectiveness in response to ongoing changes in the unregulated drug supply: “*Sometimes, I feel like smoking doesn’t hit as hard. That’s when I inject it, and it hits stronger because it goes straight into your veins*.” (Tijuana, age 26) Injecting intermittently was especially common for people using methamphetamine because methamphetamine potency varied more from day to day and the perceived discrepancy between injecting and smoking methamphetamine was larger than for fentanyl: “*Sometimes I [inject] it just because it’s a weaker strain of methamphetamine, and I need to be more awake*; *smoking is just not cutting it.”* (San Diego, age 34) These choices reflect the interplay between structural drug market conditions and individual strategies to manage drug effects.

#### 3.2.4. Individual embodied injection practices and perceptions about the necessity of injection constrained shifts from injecting to smoking

Although most participants preferred smoking, particularly for fentanyl, many described injecting as an embodied practice developed over years of use, as one noted, *“I’ve been using the needle for so long that I just prefer injecting…it’s easier for me than smoking.”* (Tijuana, age 47) Several others did not stop injecting altogether because they *“really liked shooting up… and there’s no better way of doing it than by going for the veins.”* (San Diego, age 34) Some specifically cited concerns about transitioning from injecting to smoking due to fears of not being able to manage withdrawal or pain if the effects of smoking were not intense enough to match their tolerance to drug use: *“Once you’re addicted like this, if I only smoked, my body wouldn’t react the same way.”* (Tijuana, age 48) These accounts suggest that, despite increasingly common preferences for smoking fentanyl (and, to a lesser extent, methamphetamine), most participants injected intermittently rather than exclusively smoking because injection “*hits you harder*” (Tijuana, age 55) and provides more reliable relief.

#### 3.2.5. Structural and community-level gaps in access to safer smoking equipment precipitated injection and improvised equipment use

Across sites, limited access to free safer smoking equipment meant that several participants had to inject when, *“you don’t have a pipe [or] any foil, [and] you just do a shot.”* (San Diego, age 37) However, access to sterile drug use equipment differed sharply by site.

In San Diego, no participants described needing to borrow or reuse syringes because they could access them through SSPs. Instead, most relied on their peers for sterile drug use equipment or opted for using aluminum foil, which is “*so cheap and easy,*” (San Diego, age 37) while they waited to afford or gain access to smoking equipment instead of injecting.

Conversely, in Tijuana, many participants needed to borrow syringes or use those *“[found on] the ground”* (age 34) to inject because they did not have free access to syringes *or* smoking equipment. Many Tijuana participants described using improvised or found materials, including household products, to inject or smoke, as one explained, *“Oh, yes. [I find them] on the street, [like] an aluminum can [for] either smoking or injecting.”* (age 51) Unlike in San Diego where participants often opted to use aluminum foil when they did not have access to safer smoking equipment, *“aluminum foil is harder to get”* (age 44) in Tijuana. Limited access to both syringes and smoking materials meant that participants had to borrow, reuse, or improvise equipment, sometimes using whichever route matched what was immediately available: *“I was in withdrawal coming out of jail, and I picked up used syringes to inject…in withdrawal, you pick up whatever you find…nothing else matters; all you want is relief.”* (Tijuana, age 34)

## 4. Discussion

In this binational context, where rapid declines in injecting opioids and stimulants have been observed (Eger et al., 2024a), we found that shifts in routes of administration were often intentional but partial and substance specific. Participants attributed increasing smoking to desire to avoid fentanyl-related harms, lower stigma and easier concealment of smoking, and drug market and polysubstance use dynamics; constraints on shifts toward smoking included embodied injection practices, perceived need for stronger effects or withdrawal relief, and inconsistent smoking equipment access.

Smoking fentanyl was overwhelmingly described as a deliberate strategy to reduce individual-level harms associated with injection, particularly overdose, skin and soft tissue infections, and vein damage. This aligns closely with qualitative findings from San Francisco and Vancouver, where shifts toward smoking opioids were framed as a pragmatic response to health issues related to drug injection and changes in the unregulated drug supply (Kral et al., 2021; Bonn et al., 2025; Ciccarone et al., 2024). In our study, participants emphasized that injecting fentanyl was often unnecessary given its potency, and, for some, viewed as too dangerous to justify. The pharmacologic properties of fentanyl (i.e., rapid onset, high potency, short duration) appeared to both motivate smoking and discourage injection (Ciccarone et al., 2017). Importantly, emerging quantitative work likewise links smoking, relative to injecting, with lower levels of non-fatal overdose, skin infections (Megerian et al., 2023), and all-cause mortality (Karandinos et al., 2025), while smoking is not without risk (Englander et al., 2026) including fatal overdose (Tanz et al., 2024). In the context of this emerging literature, our findings reinforce that transitions to smoking were often intentional, harm-reducing adaptations in the face of an increasingly complex drug supply. However, for some, injection remained a regular practice, especially for people using methamphetamine. This reflects longstanding literature on injection as a culturally embedded practice that is sometimes integral to survival (Harris et al., 2020; GRIFFITHS et al., 1994), and complicates narratives of homogeneous transitions from injecting to smoking. Thus, even within a broader shift toward smoking, injection remained relevant, functional, and, at times, preferred.

Our findings suggest important interactions across the interpersonal- and community-levels of SEM. Shifts from injecting to smoking were driven in part by changing perceptions of risk and stigma within interpersonal networks. Injecting fentanyl was viewed as especially dangerous due to its association with overdose and visible health complications (i.e., injection stigmata). In contrast, smoking was perceived as less visible, less likely to attract attention, and more socially acceptable, which may be more compatible with daily survival as policing and involuntary displacement of people experiencing homelessness have intensified (Barocas et al., 2023; Goldshear et al., 2023). These dynamics sometimes led participants to inject in private settings, which may increase fatal overdose risk by reducing the likelihood of timely intervention (e.g., by administering naloxone) (Eger et al., 2024b). While findings mirror prior work documenting the role of stigma and social norms in shaping routes of administration (Bonn et al., 2025; Harris et al., 2020; Bravo et al., 2003), they suggest that overdose risk and criminalization may be intensifying injection-related stigma in the fentanyl era, particularly when combined with community-level conditions such as policing and housing instability.

Shifts from injecting to smoking were closely tied to structural changes in the unregulated drug supply—particularly the increasing presence of fentanyl (Strathdee et al., 2010). However, participants’ experiences with these changes in the drug supply differed across sites and corresponded to some differences in drug use practices. In San Diego, participants rarely described ongoing heroin injection, instead characterizing a near-complete shift toward fentanyl smoking. This pattern was described as widespread and normative, reflecting a broader transformation in opioid use practices documented across the U.S. West Coast (Kral et al., 2021; Parent et al., 2021; Lipira et al., 2026). Conversely, participants in Tijuana more commonly described continued heroin injecting, including use of “China white,” a powder form of heroin often containing fentanyl (Friedman et al., 2022a; Fleiz et al., 2020) and associated with increased overdose risk (Bailey et al., 2022). These differences in behaviors may reflect variation in local drug market dynamics, including the timing and visibility of fentanyl penetration into the unregulated drug supply. Still, in this context, we found that participants framed route decisions as pragmatic responses to their immediate experiences with the drug supply, injecting when drug quality was poor or when potency required more efficient delivery; and smoking when potency was too high. Notably, while fentanyl injection was often described as undesirable, nearly one-third of participants reported injecting it in the past month, underscoring the persistence of injection within broader shifts toward smoking.

Inconsistent access to safer smoking equipment, predominantly glass pipes, was a key constraint on shifts toward smoking (Eger et al., 2025). Across sites, limited access to this equipment sometimes precipitated injection. While the relatively robust harm reduction infrastructure in San Diego reduced syringe reuse and borrowing, allowing people to avoid many injection-related harms, limited access to sterile syringes in Tijuana led participants to reuse syringes, borrow from peers, or even improvise injection equipment. These findings underscore that transitions to smoking do not automatically translate into reductions in injection-related harm; their protective potential depends on access to sterile drug use equipment, especially in contexts with insufficient saturation of sterile syringes (Bluthenthal et al., 2007). Although smoking may reduce exposure to blood-borne infections and injection-related wounds, shared and improvised smoking equipment may introduce other health harms. For example, residual fentanyl on pipes or foil can remain bioactive (Ciccarone et al., 2024), potentially introducing overdose risk for individuals with low opioid tolerance or when exposure is unintentional. Such findings highlight the need to expand access to safer smoking equipment while maintaining syringe coverage, particularly in settings with limited harm reduction infrastructure such as Tijuana (Bejarano Romero et al., 2023). Research on the implementation determinants of harm reduction services in the SDTBR is needed to understand how this context impacts service reach and accessibility, and future longitudinal research could help disentangle how changing drug market conditions and other structural determinants (e.g., harm reduction infrastructure) interact to shape transitions between injecting and smoking over time.

Several limitations should be considered when interpreting our findings. First, we interviewed a relatively small sample of PWID drawn from an existing cohort in the SDTBR, which may not fully represent the broader population of PWID in either city or other settings, despite our use of purposive sampling. Second, findings reflect self-reported experiences that may be subject to social desirability bias, though all interviewers were trained to minimize this bias. Third, relatively low access to drug checking services in both settings may have led participants to misclassify fentanyl use as heroin use, especially in Tijuana where fentanyl penetrated heroin supplies later and more slowly than San Diego (Friedman et al., 2022b). Finally, we relied on retrospective accounts of shifts toward smoking; prospective quantitative research could help illuminate the frequency and durability of these shifts.

## 5. Conclusion

In this qualitative study from the SDTBR, shifts from injecting to smoking fentanyl and methamphetamine were largely intentional harm reduction strategies in response to increasing fentanyl presence and escalating injection-related harms. However, decisions about routes of administration were shaped not only by individual preference, but also by interpersonal dynamics, community environments, and structural conditions, including drug market volatility and access to harm reduction resources. Expanding access to comprehensive harm reduction services, including safer smoking equipment, will be critical to supporting safer consumption practices amid an increasingly unpredictable drug supply.

## Figures and Tables

**Table 1:** Sociodemographic characteristics and substance use behaviors of interviewees, October–December 2024.

	San Diego (n = 18; 50%)	Tijuana (n = 18; 50%)	Overall (N = 36; 100%)
**Sociodemographic characteristics**			
Median age in years (IQR)	37 (34, 49)	43 (34, 51)	40 (34, 51)
Assigned female sex at birth	7 (39%)	7 (39%)	14 (39%)
Hispanic/Latino/Mexican	5 (28%)	16 (89%)	21 (58%)
Monthly income < 500 USD	9 (50%)	17 (94%)	26 (72%)
Completed at least secondary school education	10 (56%)	14 (78%)	24 (67%)
Incarceration history of jail or prison	15 (83%)	9 (50%)	24 (67%)
Housing status (past 6 months)			
Unsheltered homelessness	15 (83%)	6 (33%)	21 (58%)
Sheltered homelessness	0 (0%)	7 (39%)	7 (19%)
Not experiencing homelessness	3 (17%)	5 (28%)	8 (22%)
**Substance use behaviors (past 30 days)**			
Injected heroin	7 (39%)	13 (72%)	21 (58%)
Injected fentanyl	7 (39%)	5 (28%)	12 (33%)
Injected methamphetamine	14 (78%)	6 (33%)	22 (61%)
Median days since last injection (IQR)	11 (4, 30)	1 (0, 15)	6 (0, 30)
Smoked heroin	13 (72%)	7 (39%)	20 (56%)
Smoked fentanyl	14 (78%)	4 (22%)	18 (50%)
Smoked methamphetamine	17 (94%)	16 (89%)	33 (92%)
Median days since last smoke (IQR)	0 (0, 1)	0 (0, 0)	0 (0, 0)
Injected and smoked*	13 (72%)	16 (89%)	29 (81%)
Smoked-only*	4 (22%)	2 (11%)	6 (17%)
Injected-only*	1 (6%)	0 (0%)	1 (3%)

Notes.

*= heroin, fentanyl and/or methamphetamine.

## Data Availability

Due to the sensitive nature of the data, to protect confidentiality and privacy of interviewees, raw data will not be made available.
